# Novel circular RNA circSOBP governs amoeboid migration through the regulation of the miR‐141‐3p/MYPT1/p‐MLC2 axis in prostate cancer

**DOI:** 10.1002/ctm2.360

**Published:** 2021-03-26

**Authors:** Fan Chao, Zhenyu Song, Shiyu Wang, Zhe Ma, Zhiyuan Zhuo, Ting Meng, Guoxiong Xu, Gang Chen

**Affiliations:** ^1^ Department of Urology Jinshan Hospital Fudan University Shanghai P. R. China; ^2^ Department of Surgery Shanghai Medical College Fudan University Shanghai P. R. China; ^3^ Research Center for Clinical Medicine Jinshan Hospital Fudan University Shanghai P. R. China

**Keywords:** amoeboid migration, circRNA, circSOBP, metastasis, prostate cancer

## Abstract

**Background:**

Metastatic prostate cancer is a fatal disease despite multiple new approvals in recent years. Recent studies revealed that circular RNAs (circRNAs) can be involved in cancer metastasis. Defining the role of circRNAs in prostate cancer metastasis and discovering therapeutic targets that block cancer metastasis is of great significance for the treatment of prostate cancer.

**Methods:**

The circSOBP levels in prostate cancer (PCa) were determined by qRT‐PCR. We evaluated the function of circSOBP using a transwell assay and nude mice lung metastasis models. Immunofluorescence assay and electron microscopic assay were applied to determine the phenotypes of prostate cancer cells’ migration. We used fluorescence in situ hybridization assay to determine the localization of RNAs. Dual luciferase and rescue assays were applied to verify the interactions between circSOBP, miR‐141‐3p, MYPT1, and phosphomyosin light chain (p‐MLC2).

**Results:**

We observed that circSOBP level was significantly lower in PCa specimens compared with adjacent noncancerous prostate specimens, and was correlated with the grade group of PCa. Overexpression of circSOBP suppressed PCa migration and invasion *in vitro* and metastasis *in vivo*. CircSOBP depletion increased migration and invasion and induced amoeboid migration of PCa cells. Mechanistically, circSOBP bound miR‐141‐3p and regulated the MYPT1/p‐MLC2 axis. Moreover, the depletion of MYPT1 reversed the inhibitory effect of circSOBP on the migration and invasion of PCa cells. Complementary intronic Alu elements induced but were not necessary for the formation of circSOBP. The nuclear export of circSOBP was mediated by URH49.

**Conclusion:**

Our results suggest that circSOBP suppresses amoeboid migration of PCa cells and inhibits migration and invasion through sponging miR‐141‐3p and regulating the MYPT1/p‐MLC2 axis.

## Graphical abstract

1. CircSOBP is downregulated in prostate cancer.
2. CircSOBP governs amoeboid migration and metastasis of prostate cancer through the miR‐141‐3p/MYPT1/p‐MLC2 axis.
3. The nuclear export of circSOBP is mediated by URH49.

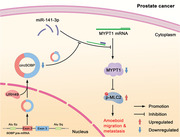

**Abbreviations**
ANP: adjacent noncancerous prostate
AUC: area under the curve
cDNA: complementary DNA
circRNA: circular RNA
DAPI: 4’,6‐Diamidino‐2‐phenylindole
EMT: epithelial‐mesenchymal transition
FBS: fetal bovine serum
FISH: fluorescence in situ hybridization
gDNA: genomic DNA
HRP: horseradish peroxidase
miRNAs: micro RNA
MLC2: myosin light chain 2
MYPT1: myosin phosphatase target subunit 1
PBS: phosphate‐buffered saline
PCa: prostate cancer
PFA: paraformaldehyde
PVDF: polyvinylidene fluoride
qRT‐PCR: quantitative real‐time polymerase chain reaction
RNA: ribonucleic acid
RNase R: ribonuclease R
ROC curve: receiver operating characteristic curve
siRNA: small interfering RNA
SOBP: sine oculis binding protein homolog
UAP56: 56 kDa U2AF65‐associated protein
URH49: UAP56‐related helicase, 49 kDa
WT: wild type
3’‐UTR: 3’‐untranslated region

## 1 BACKGROUND

Prostate cancer (PCa) accounts for 21% of all cancers and 10% estimated cancer death in men in 2020,^1^ is one of the most frequently diagnosed malignant tumors among men worldwide. Metastatic PCa is a fatal disease despite multiple new approvals in recent years, while its treatment is getting increasingly difficult.^2^, ^3^ The 5‐year survival rate of PCa patients with the localized or regional disease was more than 90%, while the 5‐year survival rate of those with the distant disease was about 30%.^3^ Investigating the molecular mechanism of cancer metastasis and discovering therapeutic targets that block cancer metastasis is of great significance for the cure of PCa.

The cellular and molecular mechanisms of cancer metastasis are incompletely understood. The migration of invasive cells consists of mesenchymal migration and amoeboid migration.^4^, ^5^ Mesenchymal migration is regulated by the epithelial‐mesenchymal transition (EMT) program. The amoeboid migration is less clear, in which cancer cells show morphological plasticity, enabling them to squeeze through interstices in the extracellular matrix. Cells undergoing amoeboid migration are accompanied by the formation of amoebic protrusions, efficiently invading adjacent tissues and spreading to distal organs through the amoeboid migration.^6^, ^7^ Morphology of cell pseudopods, cell roundness, and increased phosphomyosin light chain 2 (p‐MLC2) can define the amoeboid migration of cancer cells.^7^, ^8^, ^9^

Circular RNAs (circRNAs) are single strand, covalently closed RNAs that are generated by backsplicing of pre‐mRNAs and other transcripts.^10^ Flanking intronic complementary sequences, including Alu elements, could lead to backsplicing and the formation of circRNAs.^11^, ^12^, ^13^, ^14^ The majority of circRNAs that are generated from exonic sequences (exonic circRNAs) are located in the cytoplasm, although the backsplicing of exons occurred in the nucleus.^15^ A previous study identified key factors that mediated the nuclear export of circRNAs using RNAi screening.^16^ This work suggested that the depletion of 56 kDa U2AF65‐associated protein (UAP56) led to the accumulation of long (>1200 nucleotides) circRNAs in the nucleus, while the depletion of UAP56‐related helicase, 49 kDa (URH49) caused short (<400 nucleotides) circRNAs to be enriched in the nucleus. CircRNAs are initially proposed to be “noise” generated by aberrant splicing.^17^ However, recent progress has uncovered the biogenesis and biological function of circRNAs in healthy tissues and disease.^14^, ^18^ Recent studies revealed that circRNAs can be involved in cancer metastasis.^19^, ^20^ CircRNAs regulate cancer metastasis through interacting with EMT transcription factors,^21^, ^22^ TGF‐β‐related EMT,^23^ Wnt pathway,^24^, ^25^ cell adhesion molecules,^26^ and tumor microenvironment.^18^ However, circRNAs have not been investigated in regulating the amoeboid migration in cancer cells.

In the present study, we characterized a new circRNA in PCa. The novel circRNA is derived from the exon 2 and exon 3 of the sine oculis binding protein homolog (SOBP) gene, thereby being named circSOBP (genomic location: chr6: 107824860–107827631; circBase^27^ ID: hsa_circ_0001633). We investigated the function of circSOBP to the amoeboid migration of PCa through *in vitro* experiments. We also examined whether the loss of circSOBP induced amoeboid migration by sponging miR‐141‐3p and regulating myosin phosphatase target subunit 1 (MYPT1)/p‐MLC2 axis in PCa.

## 2 MATERIALS AND METHODS

### 2.1 Patients and sample preparations

We acquired PCa and adjacent noncancerous prostate (ANP) tissue specimens from 56 patients during radical prostatectomy at Jinshan Hospital, Fudan University from February 2017 to March 2018. The diagnosis was validated by two independent pathologists. All tissues were stored at −80°C immediately after surgical removal. The Ethics Committee of Jinshan Hospital, Fudan University approved the present study. We obtained written consent from each patient.

### 2.2 Cell lines

The HEK293T, RWPE‐1, DU145, PC‐3, LNCap, and 22Rv1 cell lines were obtained from FuHeng Cell Center (FuHeng, Shanghai, China). DU145 and HEK293T were maintained in DMEM‐4.5 g/L glucose (Gibco, Suzhou, Jiangsu, China). PC‐3 was cultured in DMEM/F12 (HyClone, Logan, Utah, USA). LNCap and 22Rv1 were maintained in RPMI‐1640 (Gibco). All media were supplemented with 10% fetal bovine serum (FBS) (BI, Beit Haemek, Israel), 100 U/mL penicillin, and 100 μg/mL streptomycin. RWPE‐1 was cultured in Prostate Epithelial Cell Medium (ScienCell, San Diego, CA, USA). All cell lines were cultured at 37°C containing 5% v/v CO_2_.

### 2.3 RNA interference (RNAi)

An appropriate number of cells were planted 24 h before transfection so that the monolayer cell density reached 60% at the time of transfection. Scramble small interfering RNA (siRNA) and specific siRNAs (synthesized by GenePharma, Shanghai, China) were transfected using X‐tremeGENE siRNA Transfection Reagent (Roche, Mannheim, Germany). The siRNAs were transfected at 50 pmol/mL. Sequences of the siRNAs were available in Supporting information Table S1.

### 2.4 CircRNA expressing lentivirus production and infection

The circSOBP expressing lentiviral vector pLCDH‐cir was obtained from GeneSeed (Guangzhou, Guangdong, China). We obtained the pSPAX2 and pMD2.G plasmids from Hanbio (Shanghai, China). For lentiviral production, we transfected pLCDH‐cir, pSPAX2, pMD2.G plasmids into HEK293T cells with LipoD293 DNA transfection reagent (SignaGene Laboratories, Rockville, Maryland, USA) following the manufacturer's protocol. We collected the viral supernatant at 1, 2, and 3 days after transfection and removed the cell debris by centrifuging at 1000 *g* for 5 min.

For lentiviral transduction, cells should be seeded on a 24‐well plate 12 to 24 h before infection so that the monolayer cell density reached 30‐50% at infection. The culture medium was replaced with a 1:1 diluted viral supernatant and cultured overnight. For optimal infection, cells were infected with 10 μg/mL hexadimethrine bromide. After 72 h infection, cells were cultured with 3 μg/mL puromycin to kill noninfected cells.

### 2.5 Isolation of nuclear and cytoplasmic RNA, total RNA, and micro RNA (miRNA) extraction, and quantitative real‐time polymerase chain reaction (qRT‐PCR)

The nuclear and cytoplasmic RNA was isolated and purified using Cytoplasmic & Nuclear RNA Purification Kit (Norgen Biotek, Thorold, Ontario, Canada). Total RNA was extracted using RNA‐Quick Purification Kit (Yishan, Shanghai, China). Micro RNA (miRNAs) were extracted and purified using miRNA Isolation Kit with Spin Column (Beyotime, Shanghai, China). RNA samples were quantified and qualified using NanoDrop (Thermo Fisher Scientific, Waltham, MA, USA). For reverse transcription of total RNA, 500 ng RNA was reverse transcribed in a 10 μL system using PrimeScript RT Master Mix (Takara, Shiga, Japan). Reverse transcription of miRNAs was conducted using Mir‐X miRNA First‐Strand Synthesis Kit (Takara). Complementary DNA (cDNA) was diluted with diethylpyrocarbonate‐treated water. PCR was conducted in triplicate on the ABI 7300 Real‐Time PCR system (Thermo Fisher Scientific) using BeyoFast SYBR Green qPCR Mix (Beyotime) following the manufacturer's protocol. The cycling parameters were: 95°C for 2 min initially, 40 cycles of 95°C for 15 s followed by 60°C for 30 s. We analyzed the PCR results using the ΔΔCT method.^28^ Sequences of primers are available in Supporting information Table S2.

### 2.6 Cell viability, colony formation, and apoptosis assay

We planted an appropriate number of cells in 96‐well plates and measured viability using Cell Counting Kit‐8 (Dojindo, Kumamoto, Japan) every 24 h. All measurements were performed at least three times.

Colony formation was determined by seeding 1000 cells in a 6‐well plate. Ten days after cultivation, cells were fixed using 4% paraformaldehyde (PFA) for 15 min and stained using the 1% crystal violet solution for another 30 min. Finally, the colonies were photographed and counted.

Apoptosis was analyzed using One‐Step TUNEL Apoptosis Assay Kit (Beyotime). Images were photographed using a fluorescence inverted microscope (Olympus, Tokyo, Japan).

### 2.7 Migration and invasion assays

Transwell permeable supports with 8 μm pore (Corning, New York City, USA) and 24‐well plates were used to conduct migration and invasion assays.

For invasion assay, the upper chambers were coated using diluted Matrigel (Corning) at 37°C for 4 h before seeding cells. For the migration assay, the upper chambers were not coated. An appropriate number of cells were seeded with an FBS‐free medium in the upper chamber of the Transwell permeable support. For DU145, we seeded 6 × 10^4^ cells/well for “NC versus circSOBP” groups and 3 × 10^4^ cells/well for “si‐NC versus si‐circSOBP” groups, respectively. For PC‐3, we seeded 8 × 10^4^ cells/well for “NC versus circSOBP” groups and 6 × 10^4^ cells/well for “si‐NC versus si‐circSOBP” groups, respectively. The lower chamber was filled with 400 μL complete medium supplied with 100 μL FBS. Appropriate subsequent culture time varies from cell lines (24 h for DU145 and 48 h for PC‐3). The migrated cells were fixed using 4% PFA followed by staining using 1% crystal violet solution. We counted the migrated cells from three random fields.

### 2.8 Generation of a nude mouse model

Briefly, 1 × 10^7^ DU145 cells stably overexpressing circSOBP and its control cells were injected into the tail vein of nude mice (n = 10 per group). Eight weeks after injection, the nude mice were sacrificed and infused with formalin. Subsequently, the lungs of the nude mice were resected and fixed with formalin. After counting the metastatic nodules, we embedded the lungs in paraffin and made paraffin sections. Finally, the paraffin sections were stained using hematoxylin and eosin staining.

### 2.9 Dual‐luciferase assay

The sequences of the wild‐type circSOBP and *MYPT1* 3’‐untranslated region (UTR), which were predicted to target miR‐141‐3p or their mutants, were synthesized and cloned into pmirGLO vector (Promega, Madison, Wisconsin, USA) by Genewiz (Shanghai, China) between the NheI and XhoI restriction sites. PC‐3 cells were transfected using X‐tremeGENE siRNA Transfection Reagent (Roche). The pmirGLO plasmid and miRNA mimic were transfected at 1 μg/mL and 50 pmol/mL, respectively. Luciferase activity was tested 48 h post‐transfection using Dual‐Lumi Luciferase Reporter Gene Assay Kit (Beyotime).

### 2.10 Western blot

We extracted total protein using SDS lysis buffer (Beyotime) supplemented with protease and phosphatase inhibitor cocktail (Beyotime). Total proteins were quantified using the Enhanced BCA Protein Assay Kit (Beyotime). Protein (30 μg per lane) was loaded into 12% SDS‐PAGE gel and run at 120 V for 2 h. Proteins were transferred to polyvinylidene fluoride (PVDF) membranes (Millipore, Billerica, Massachusetts, USA) at 250 mA for 1 h. PVDF membranes containing proteins were blocked by QuickBlock Blocking Buffer for Western Blot (Beyotime) at room temperature for 20 min and cut horizontally to examine multiple proteins of different sizes. Then, membranes were incubated with diluted primary antibodies (Supporting information Table S3) at 4°C overnight, followed by washing for three times. Membranes were subsequently soaked with horseradish peroxidase (HRP)‐conjugated secondary antibodies at room temperature for 1 h. Finally, blots were visualized using Immobilon Western Chemiluminescent HRP Substrate (Millipore).

### 2.11 Immunofluorescence staining

In order to observe the amoeboid features of cells, a 96‐well plate was coated with 1:20 diluted Matrigel Matrix (Corning) and incubated at 37°C overnight. Subsequently, cells were plated and cultured overnight. Cells were fixed for 15 min using 4% PFA followed by permeabilization using phosphate‐buffered saline (PBS) supplemented with 0.1% Triton X‐100 for 15 min. Subsequently, cells were blocked using PBS supplemented with 5% bovine serum albumin and 0.1% Tween20 for 1 h at room temperature. After blocking, cells were incubated with a primary antibody (Supporting information Table S3) at 4°C overnight. Then the cells were incubated with Alexa Fluor 555‐labeled secondary antibody at room temperature for 1 h. F‐actin was stained using iFluor 488‐conjugated phalloidin (Yeasen, Shanghai, China). 4’,6‐Diamidino‐2‐phenylindole (DAPI) was used to stain the nuclei. All pictures were photographed using a fluorescence inverted microscope (Olympus).

### 2.12 RNA fluorescence in situ hybridization (RNA‐FISH)

RNA‐ fluorescence in situ hybridization (FISH) assay was conducted to determine the subcellular location of circSOBP. RNA‐FISH kit and a mix of three 5’‐Cy3 labeled probes that targeted the backsplice site of circSOBP were obtained from GenePharma (Shanghai, China). We carried out the hybridization according to the manufacturer's protocol. The concentration of the probe mix was 8 μmol/L during hybridization. All pictures were obtained using a fluorescence inverted microscope (Olympus). Sequences of circSOBP probes are listed in Supporting information Table S4.

### 2.13 Cell roundness index calculation

The cell roundness index between 0.0 and 1.0 was calculated by dividing the shortest diameter by the longest diameter of each cell.^9^, ^29^ The roundness index of a perfectly round cell is 1.0.

### 2.14 Statistical analyses

We conducted all experiments at least three times. We performed statistical analyses using SPSS Statistics (IBM, Chicago, IL, USA) or Prism 8 (GraphPad Software, San Diego, CA, USA). Student's *t*‐test, one‐way ANOVA, chi‐squared test, Fisher's exact test, Mann–Whitney *U* test, and Wilcoxon matched‐pairs signed‐rank test were used for comparisons, as appropriate. The levels of significance were set at: *P < .05, **P < .01, and ***P < .001.

## 3 RESULTS

### 3.1 Characterization of circSOBP in PCa

To characterize PCa‐related circRNAs, our circRNA microarray across five pairs of ANP tissues and PCa tissues showed that a series of circRNAs were differentially expressed.^30^ CircSOBP was chosen for the present study, the reasons were: (i) The circRNA microarray showed that circSOBP (genomic location: chr6: 107824860–107827631; circBase ID: hsa_circ_0001633)^27^ was downregulated in PCa tissues. (ii) CircSOBP was of relatively high abundance, ranked 4th in PCa and 6th in ANP tissues among the 24 downregulated circRNAs (Supporting information Figure S1A); (iii) the biological function of circSOBP remained unknown.

**FIGURE 1:**
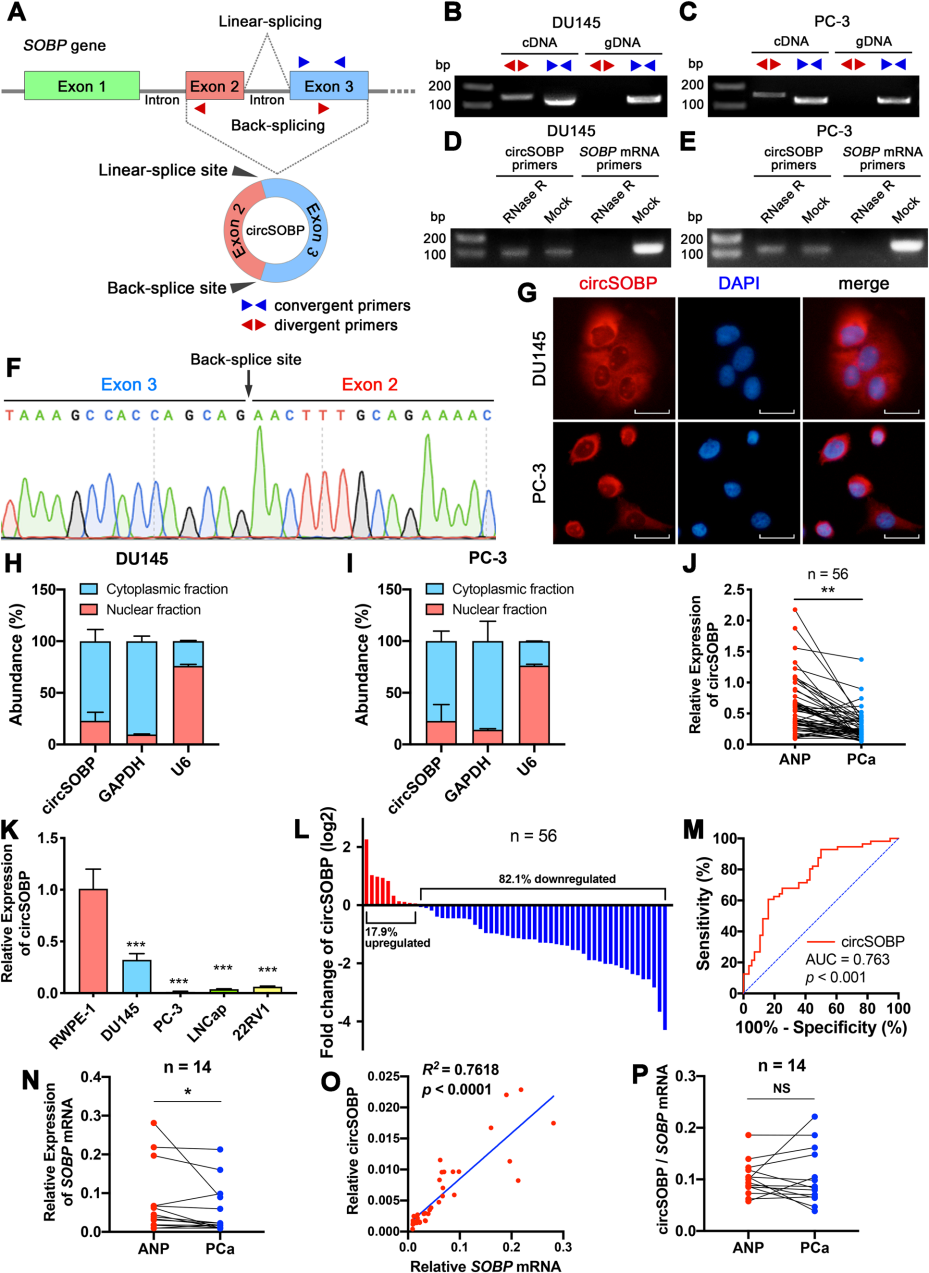
The characteristics of the circular RNA circSOBP. (A) Schematic illustration of the origin of circSOBP. Blue triangles indicate the convergent primers that amplify the linear sequence of circSOBP. Red triangles indicate the divergent primers that amplify the backsplice site of circSOBP. (B) and (C) Divergent and convergent primers were used to detect circSOBP by PCR in cDNA and gDNA of DU145 and PC‐3 cell lines. (D) and (E) CircSOBP and *SOBP* mRNA were detected using PCR in RNase R treated total RNAs of DU145 and PC‐3 cell lines. (F) Sanger sequencing of the backsplicing site of circSOBP. (G) The FISH assay was used to detect the subcellular localization of circSOBP (red), combined with nuclei staining using DAPI (blue). Scale bar, 30 μm. (H) and (I) Abundance of circSOBP in the nuclear and cytoplasmic fractions of DU145 and PC‐3 cells, analyzed using qRT‐PCR. *GAPDH* mRNA, and U6 acted as a positive control. The data are presented as the mean ± SD. (J) Expression of circSOBP in ANP and PCa tissues of 56 patients was analyzed by qRT‐PCR. **P < .01, Wilcoxon matched‐pairs signed‐rank test, n = 56. (K) Expression of circSOBP in prostate epithelial and PCa cell lines was analyzed by qRT‐PCR. The data are presented as the mean ± SD. ***P < .001 versus RWPE‐1 cells, one‐way ANOVA and Dunnett's multiple comparisons test, n = 3. (L) The percentage of upregulated or downregulated expression of circSOBP in 56 patients. (M) The ROC curve in distinguishing PCa and ANP tissues by the expression of circSOBP. (N) Expression of *SOBP* mRNA in ANP and PCa tissues of 14 patients was analyzed by qRT‐PCR. * *P* < .05, Wilcoxon matched‐pairs signed‐rank test, n = 14. (O) Scatter plot of the correlation between the expression of circSOBP and *SOBP* mRNA. Linear regression model, *R* ^2^ = 0.7618, *P* < .0001. (P) Ratio of the expression of circSOBP and *SOBP* mRNA in ANP and PCa tissues of 14 patients. Paired *t*‐test, n = 14. NS, not significant

CircSOBP is derived from exon 2 and exon 3 of the *SOBP* gene, thereby being named circSOBP (Figure 1A). The full length of circSOBP is shown in Supporting information Figure S1B. We designed divergent primers that amplified the backsplicing site of circSOBP and convergent primers that amplified the linear part of circSOBP as well as the *SOBP* gene (Figure 1A). The PCR results showed that the backsplicing site of circSOBP was only detected in cDNA but not in genomic DNA (gDNA), while the linear part of circSOBP could be detected in both cDNA and gDNA (Figure 1B,C), indicating that the sequence containing the backsplice site does not exist in the genome. In addition, circSOBP was resistant to ribonuclease R (RNase R), a 3’‐5’ exonuclease that could only digest linear transcripts but not circRNAs, indicating that circSOBP was circular (Figure 1D,E). Sanger sequencing confirmed the backsplicing site (Figure 1F). FISH (Figure 1G) indicated that circSOBP was mainly distributed in the cytoplasm. Isolation of subcellular RNAs followed by qRT‐PCR showed that circSOBP was mainly distributed in the cytoplasm (Figure 1H,I).

### 3.2 Expression of circSOBP in PCa

Specific divergent primers were used to detect circSOBP expression in PCa cells and tissue specimens. We found circSOBP level was lower in PCa tissues (Figure 1J) and PCa cell lines (Figure 1K) than that in ANP tissues and prostate epithelial cell line RWPE‐1, respectively. Among 56 paired tissues, circSOBP expression was low in 46 (82.1%) PCa tissues compared to the paired ANP tissues (Figure 1L). The receiver operating characteristic (ROC) curve indicated that the area under the curve (AUC) of circSOBP was 0.763 (cut‐off value = 0.249, sensitivity = 0.839, 1–specificity = 0.393) (Figure 1M), indicating that circSOBP was a promising biomarker for distinguishing PCa tissues.

Detailed PCa patients’ characteristics are shown in Table 1. PCa patients were divided into PCa < ANP and PCa > ANP groups based on the relative expression of circSOBP of their PCa and ANP tissues. As shown in Table 1, circSOBP levels were significantly associated with PCa patients’ Gleason score (*P* = 0.0292) and grade group (*P* = .0118).

**TABLE 1:** Correlation between circSOBP expression and clinicopathological characteristics in PCa

	Expression of circSOBP		
Parameters	PCa < ANP (n = 46)	PCa > ANP (n = 10)	*P‐*value
Age			.3150
≤67	19	6	
>67	27	4	
PSA (ng/mL)			.7287
≤14.085	22	6	
>14.085	24	4	
Gleason score			**.0292**
<7	0	2	
≥7	46	8	
Grade group			**.0118**
1	0	2	
2	12	2	
3	10	4	
4	10	0	
5	14	2	
pT stage			.8706
2	20	4	
3a	18	3	
3b	8	3	
N stage			.6691
0	39	9	
1	7	1	
Stage group			.9121
I + II	13	3	
III + IV	33	7	
Nerve invasion			.7069
Yes	26	5	
No	20	5	

ANP, adjacent noncancerous prostate; PCa, prostate cancer; PSA, prostate‐specific antigen.

In addition, we determined the correlation between circSOBP and SOBP mRNA levels in PCa. The expression of *SOBP* mRNA was lower in PCa tissue specimens than that in ANP (Figure 1N). The linear regression model suggested that the increased expression of circSOBP was associated with the increased *SOBP* mRNA (*R* ^2^ = 0.7618, *P *< .0001)(Figure 1O). However, there is no difference in the ratio of circSOBP and *SOBP* mRNA in ANP and PCa (Figure 1P).

### 3.3 CircSOBP modulation alters PCa migration and invasion *in vitro* and metastasis *in vivo*

**FIGURE 2:**
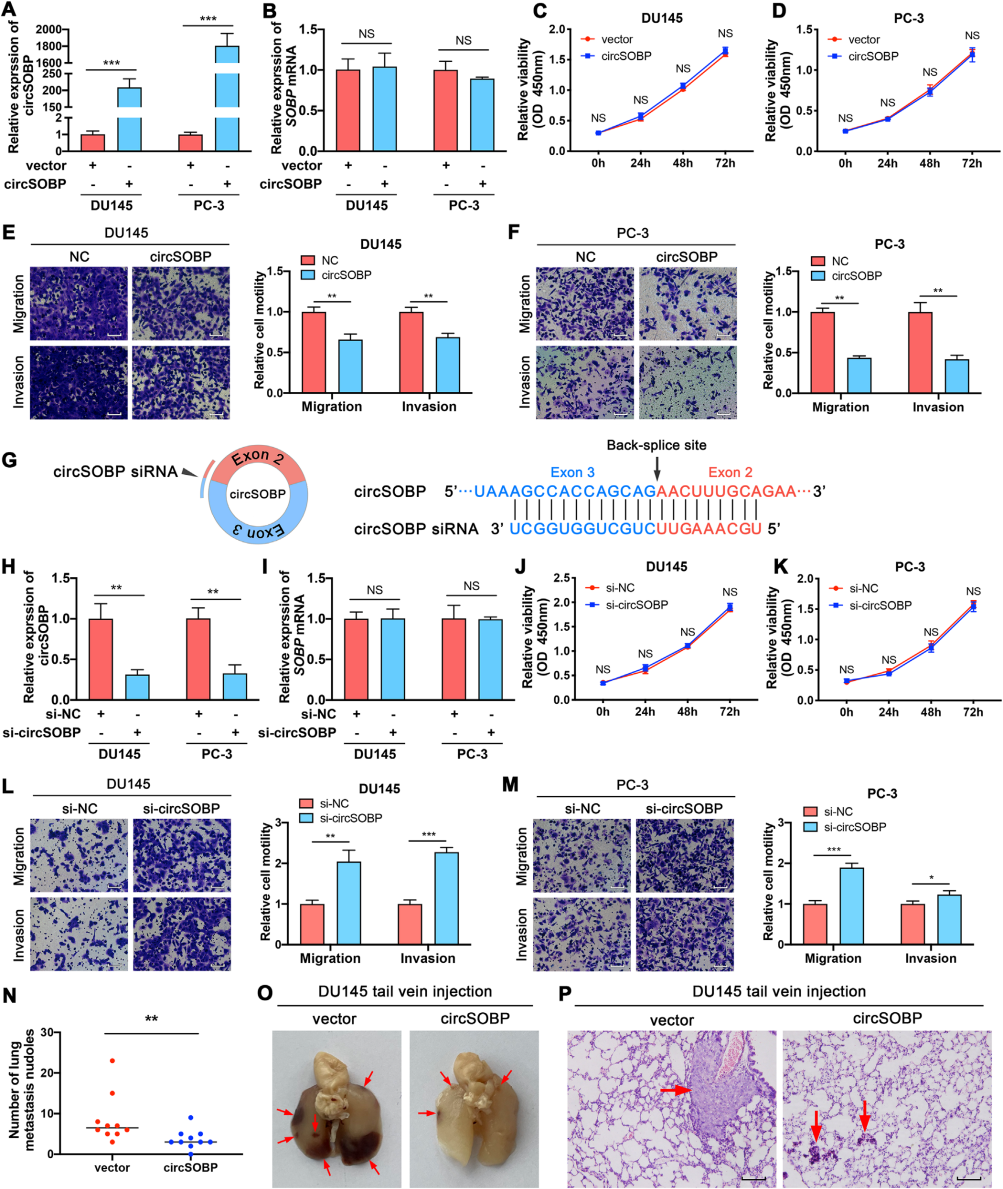
Effects of forced circSOBP expression on viability, migration, and invasion of PCa cells. (A) and (B) Expression of circSOBP and *SOBP* mRNA in circSOBP‐overexpressing DU145 and PC‐3 cells analyzed by qRT‐PCR. The data are presented as the mean ± SD. ***P < .001, Student's *t*‐test, n = 3. (C) and (D) Effect of overexpression of circSOBP on cell viability of DU145 and PC‐3 cells. The data are presented as the mean ± SD. Student's *t*‐test, n = 3. (E) and (F) Effect of overexpression of circSOBP on migration and invasion of DU145 and PC‐3 cells. Scale bar, 100 μm. The data are presented as the mean ± SD. **P < .01, Student's *t*‐test, n = 3. (G) Schematic illustration of the specific siRNA targeting the backsplicing site of circSOBP. (H) and (I) Expression of circSOBP and *SOBP* mRNA in circSOBP‐depleted DU145 and PC‐3 cells analyzed by qRT‐PCR. The data are presented as the mean ± SD. **P < .01, Student's *t*‐test, n = 3. (J) and (K) Effect of circSOBP depletion on cell viability of DU145 and PC‐3 cells. The data are presented as the mean ± SD. Student's *t*‐test, n = 3. (L) and (M) Effect of circSOBP depletion on migration and invasion of DU145 and PC‐3 cells. Scale bar, 100 μm. The data are presented as the mean ± SD. *P < .05, **P < .01, ***P < .001, Student's *t*‐test, n = 3. (N) Effect of circSOBP overexpression on the numbers of lung metastatic nodules of nude mice. Black lines in the middle of the dots depict the median. **P < .01, Mann–Whitney test, n = 10. (O) Metastatic nodules in the mouse lungs. Red arrows indicate the metastatic nodules. (P) Hematoxylin‐eosin staining of the metastatic nodules. Scale bar, 50 μm. Red arrows indicate the metastatic nodules. NC, normal control. NS, not significant

Next, circSOBP's biological functions were determined. We constructed DU145 (Supporting information Figure S2A) and PC‐3 (Supporting information Figure S2B) cells stably overexpressing circSOBP using lentiviral transduction. CircSOBP expression was significantly overexpressed (Figure 2A), without boosting the expression of *SOBP* mRNA (Figure 2B). The upregulation of circSOBP did not affect cell growth (Figure 2C,D) but inhibited migration and invasion (Figure 2E,F).

We designed siRNA targeting the backsplicing site of circSOBP (Figure 2G), which efficiently depleted circSOBP in DU145 and PC‐3 cell lines (Figure 2H), without altering the expression of *SOBP* mRNA (Figure 2I). CircSOBP depletion did not affect cell growth (Figure 2J,K). However, migration and invasion of DU145 (Figure 2L) and PC‐3 (Figure 2M) cell lines were promoted after the depletion of circSOBP expression.

We used the mouse lung metastasis model to investigate the biological function of circSOBP *in vivo*. DU145 cells stably overexpressing circSOBP formed more lung metastatic nodule than the control cells (Figure 2N). Moreover, lung metastases formed by DU145 cells stably overexpressing circSOBP were larger than those formed by control cells (Figure 2O,P).

Furthermore, we explored the effects of overexpressing or depleting circSOBP on apoptosis and colony formation of PCa cells. TUNEL staining was used to detect apoptosis. CircSOBP modulation did not induce apoptosis of DU145 (Supporting iformation Figure S2C) and PC‐3 cells (Supporting information Figure S2D). Similarly, overexpression or depletion of circSOBP did not alter colony formation of PCa cells (Supporting information Figure S2E‐G).

### 3.4 Depletion of circSOBP induces amoeboid migration of PCa cells

It is known that the mesenchymal migration of cancer cells regulated by EMT is a key process in regulating metastasis of cancer. Given circSOBP modulation alters migration and invasion of PCa cells, we assessed whether circSOBP could regulate EMT by detecting E‐cadherin and vimentin. However, overexpression of circSOBP did not alter the expression of E‐cadherin or vimentin (Supporting information Figure S3A‐E), indicating that circSOBP could not regulate the process of EMT. In addition to EMT, cancer cells can also metastasize by amoeboid migration. Therefore, we further determined whether circSOBP could alter the process of amoeboid migration of PCa cells.

**FIGURE 3:**
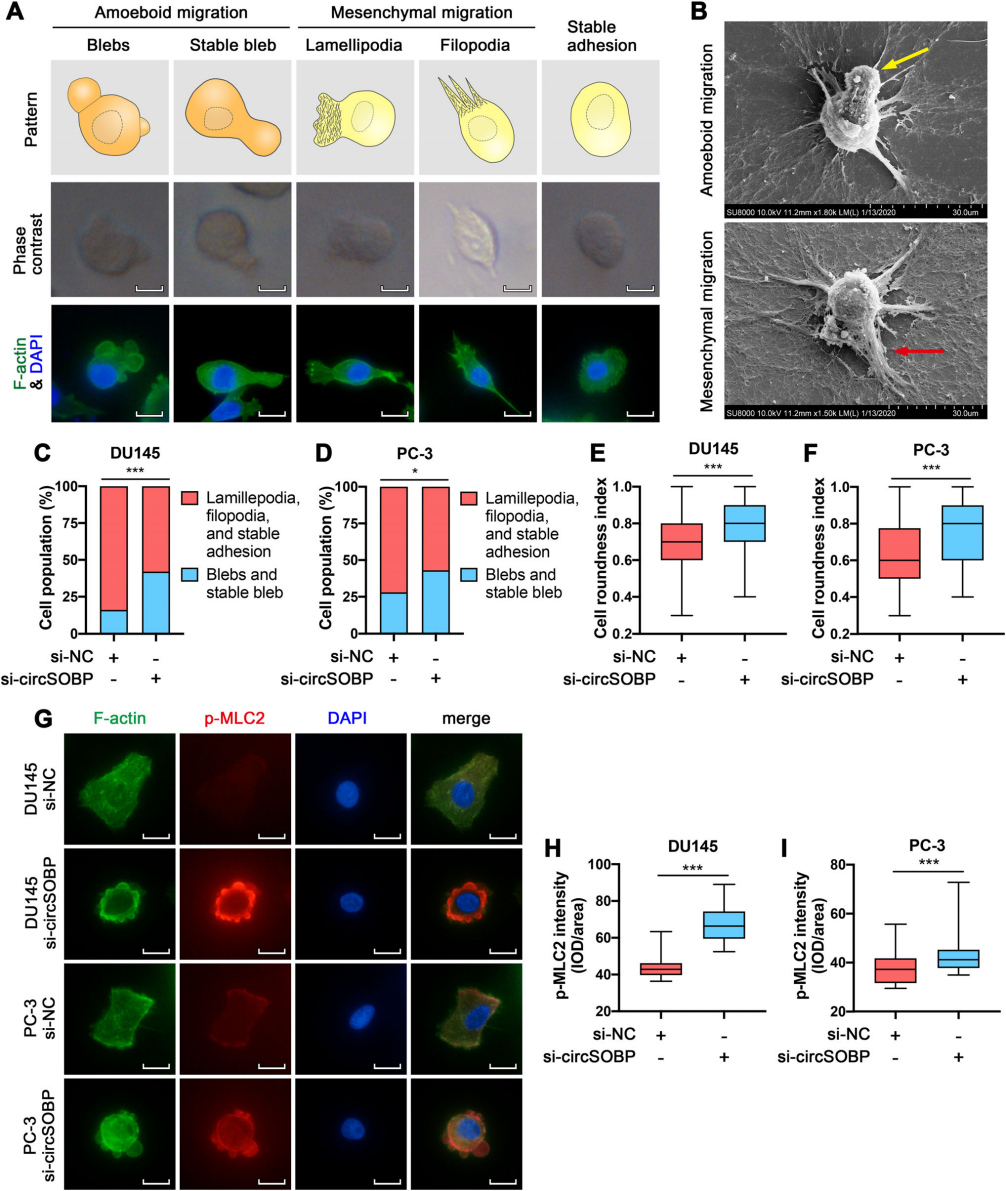
Depletion of circSOBP induces amoeboid features of PCa cells. (A) The morphology of cells undergoing mesenchymal migration or amoeboid migration on the Matrigel matrix. Scale bar, 20 μm. (B) Electron microscopic images of cells undergoing mesenchymal and amoeboid migration on the Matrigel matrix. The yellow arrow indicates a stable bleb. The red arrow indicates filopodia. Scale bar, 30 μm. (C) and (D) The percentage of circSOBP‐depleted DU145 and PC‐3 cells undergoing different phenotypes of migration on the Matrigel matrix compared with control cells. *P < .05, ***P < .001, Fisher's exact test. (E) and (F) Cell roundness of circSOBP‐depleted DU145 and PC‐3 cells. Boxplots depict median, 25th and 75th percentile, min‐max whiskers. ***P < .001, Mann–Whitney test, n = 3. (G) The morphology of circSOBP‐depleted DU145 and PC‐3 cells analyzed using immunofluorescence assay, representative images. Scale bar, 20 μm. (H) and (I) Quantification of p‐MLC2 expression from immunofluorescence assay. Boxplots depict median, 25th and 75th percentile, min‐max whiskers. ***P < .001, Mann–Whitney test, n = 3. NC, normal control

Next, we attempted to induce amoeboid migration of PCa cells by depleting circSOBP using RNA interference. The migration of PCa cells on the Matrigel matrix includes mesenchymal migration and amoeboid migration. We observed five migration phenotypes of PCa cells using a phase‐contrast microscope or fluorescence assay: cells with blebs, stable bleb, lamellipodia, filopodia, or stable adhesion (Figure 3A). Among them, cells with blebs and stable bleb are phenotypes of amoeboid migration, while cells with lamellipodia and filopodia are phenotypes of mesenchymal migration.^7^, ^9^, ^31^ Stable adhesion refers to cells that are stationary and morphologically unchanging. The morphology of cell pseudopods was also identified by electron microscopy (Figure 3B). The incidence of blebs and stable blebs was more predominant in circSOBP depleted DU145 (Figure 3C) and PC‐3 (Figure 3D) cells. In addition, roundness, morphology, and increased p‐MLC2 are characteristics of amoeboid migration.^8^, ^9^ We found that circSOBP depleted cells were rounder by calculating the roundness index (Figure 3E,F). The morphology and p‐MLC2 expression of circSOBP‐depleted DU145 and PC‐3 cells cultured on Matrigel matrix were analyzed using immunofluorescence assay (Figure 3G), indicating that p‐MLC2 was increased in circSOBP depleted cells (Figure 3H,I).

### 3.5 CircSOBP regulates MYPT1/p‐MLC2 axis by sponging miR‐141‐3p

**FIGURE 4:**
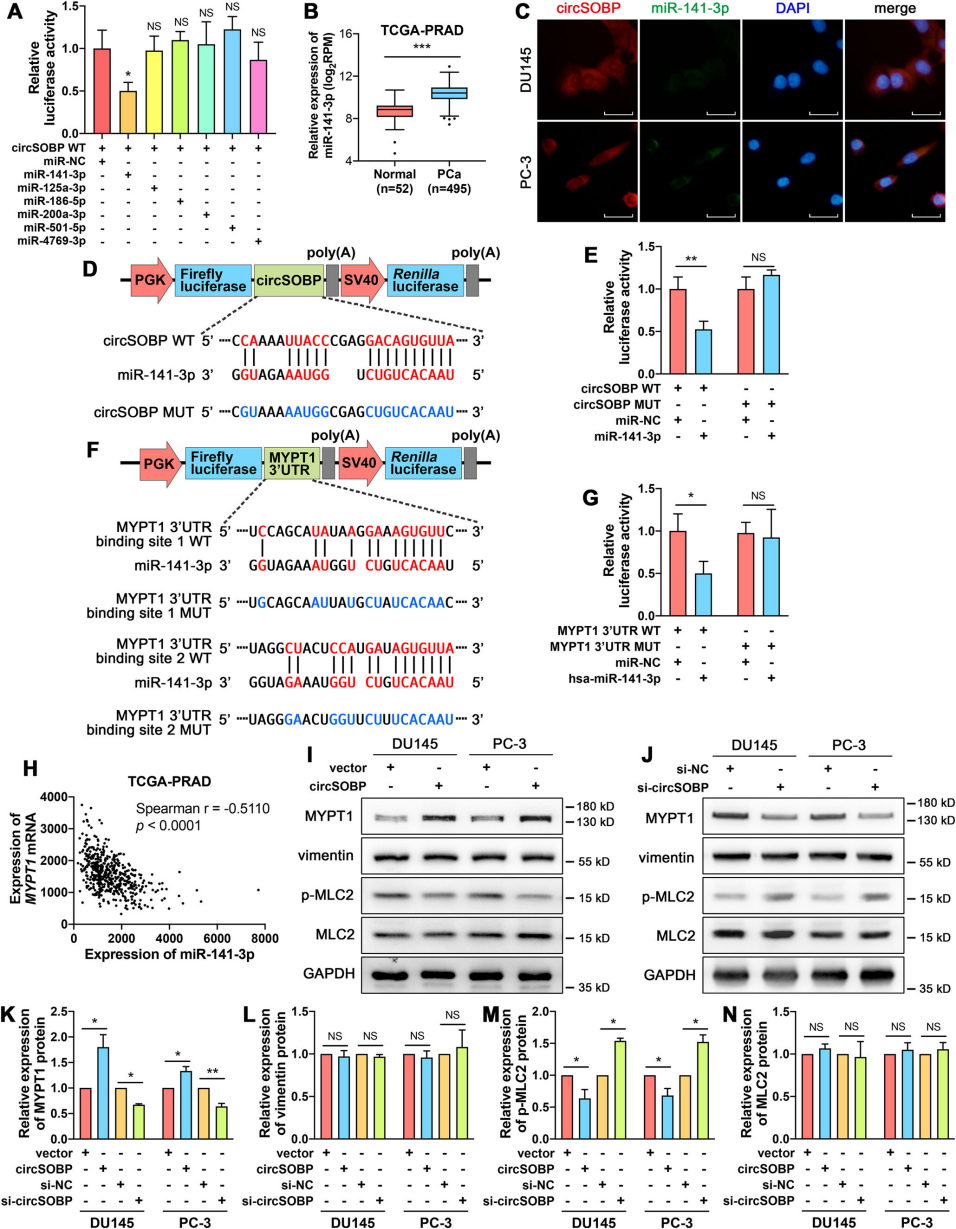
CircSOBP regulates the MYPT1/p‐MLC2 axis by sponging miR‐141‐3p. (A) Dual‐luciferase assay was used to identify the direct interaction between circSOBP and six predicted miRNAs. The data are presented as the mean ± SD. *P < .05 versus miR‐NC, one‐way ANOVA and Dunnett's multiple comparisons test, n = 3. (B) Expression of miR‐141‐3p in PCa and normal tissues in TCGA database. Boxplot depicts median, 25th and 75th percentile, min‐max whiskers. ***P < .001, Mann–Whitney test. (C) The FISH assay was used to identify the subcellular colocalization of circSOBP (red) and miR‐141‐3p (green), combined with nuclei staining using DAPI (blue). Scale bar, 30 μm. (D) Wild‐type and mutated binding site of miR‐141‐3p in circSOBP. (E) Dual‐luciferase assay was used to identify the direct interaction between circSOBP and miR‐141‐3p. The data are presented as the mean ± SD. **P < .01, Student's *t*‐test, n = 3. (F) Binding sites of miR‐141‐3p and their mutants in 3’UTR of *MYPT1* mRNA. (G) Dual‐luciferase assay was used to identify the direct interaction between the 3’‐UTR of *MYPT1* mRNA and miR‐141‐3p. The data are presented as the mean ± SD. *P < .05, Student's *t*‐test, n = 3. (H) Correlation between the expression of miR‐141‐3p and MYPT1 mRNA in the TCGA‐PRAD database. (I) Effect of circSOBP overexpression on the expression of related proteins of DU145 and PC‐3 cells. (J) Effect of circSOBP depletion on the expression of related proteins of DU145 and PC‐3 cells. (K)‐(N) Densimetric analysis of the blots in (I) and (J), n = 3 independent experiments, GAPDH was used as the loading control. The data are presented as the mean ± SD. *P < .05, **P < .01, Student's *t*‐test. MUT, mutant. NC, normal control. NS, not significant. WT, wild type

We further explored the mechanism by which circSOBP regulates the amoeboid migration of PCa. Previous studies have revealed that circRNAs could sponge miRNAs and abrogate their functions.^14^, ^32^ We predicted six potential miRNAs sponged by circSOBP using RegRNA 2.0 database.^33^ Next, we used a dual‐luciferase assay to determine the interaction between circSOBP and miRNAs, showing that circSOBP could bind miR‐141‐3p (Figure 4A). However, circSOBP overexpression did not alter the expression of the predicted miRNAs (Supporting information Figure S4). MiR‐141‐3p was upregulated in PCa (n = 52) compared with normal specimens (n = 495) (Figure 4B). RNA FISH assay indicated the colocalization of circSOBP and miR‐141‐3p in DU145 and PC‐3 cells (Figure 4C). To further verify whether circSOBP could sponge miR‐141‐3p, dual‐luciferase plasmids with wild‐type and mutant circSOBP sequences were constructed (Figure 4D). MiR‐141‐3p was able to suppress the luciferase activity in cells with wild‐type circSOBP but not in the cells with mutant circSOBP (Figure 4E).

We predicted MYPT1 as the downstream target of circSOBP/miR‐141‐3p, the reasons were as follows: (i) miR‐141‐3p targeted 3’UTR of *MYPT1* mRNA in accordance with the prediction of the ENCORI database (http://starbase.sysu.edu.cn), which were derived from CLIP‐seq data^34^; (ii) MYPT1 was downregulated in PCa according to the data from the Cancer Genome Atlas (TCGA) Research Network (https://www.cancer.gov/tcga), which is consistent with predicted expression trend of our study; (iii) cells lacking MYPT1 exhibit a hyperblebbing phenotype, which lead to amoeboid behaviors^35^; (iv) amoeboid migration requires a high level of p‐MLC2,^8^, ^9^ while MYPT1 mediates dephosphorylation of p‐MLC2.^36^, ^37^, ^38^

Dual‐luciferase plasmids with wild‐type and mutant *MYPT1* mRNA 3’UTR were used to verify the interaction between miR‐141‐3p and *MYPT1* 3’UTR (Figure 4F). We confirmed that miR‐141‐3p could bind to *MYPT1* 3’UTR (Figure 4G). A negative correlation was found between miR‐141‐3p and *MYPT1* mRNA (Spearman *r* = −0.5110, *P* < .0001) in PCa (Figure 4H) according to the TCGA data.

Besides, we found that overexpression of circSOBP could upregulate the expression of MYPT1 and downregulate the expression of p‐MLC2, but could not alter the expression of vimentin or total MLC2 (Figure 4I, K‐N). At the same time, depletion of circSOBP could downregulate MYPT1 and upregulate p‐MLC2 without altering vimentin or total MLC2 (Figure 4J, K‐N). Overexpression or depletion of circSOBP did not alter the expression of MLC2 mRNA (Supporting information Figure S3F,G).

### 3.6 Depletion of MYPT1 reverses the inhibitory effect of circSOBP on migration and invasion of PCa cells

**FIGURE 5:**
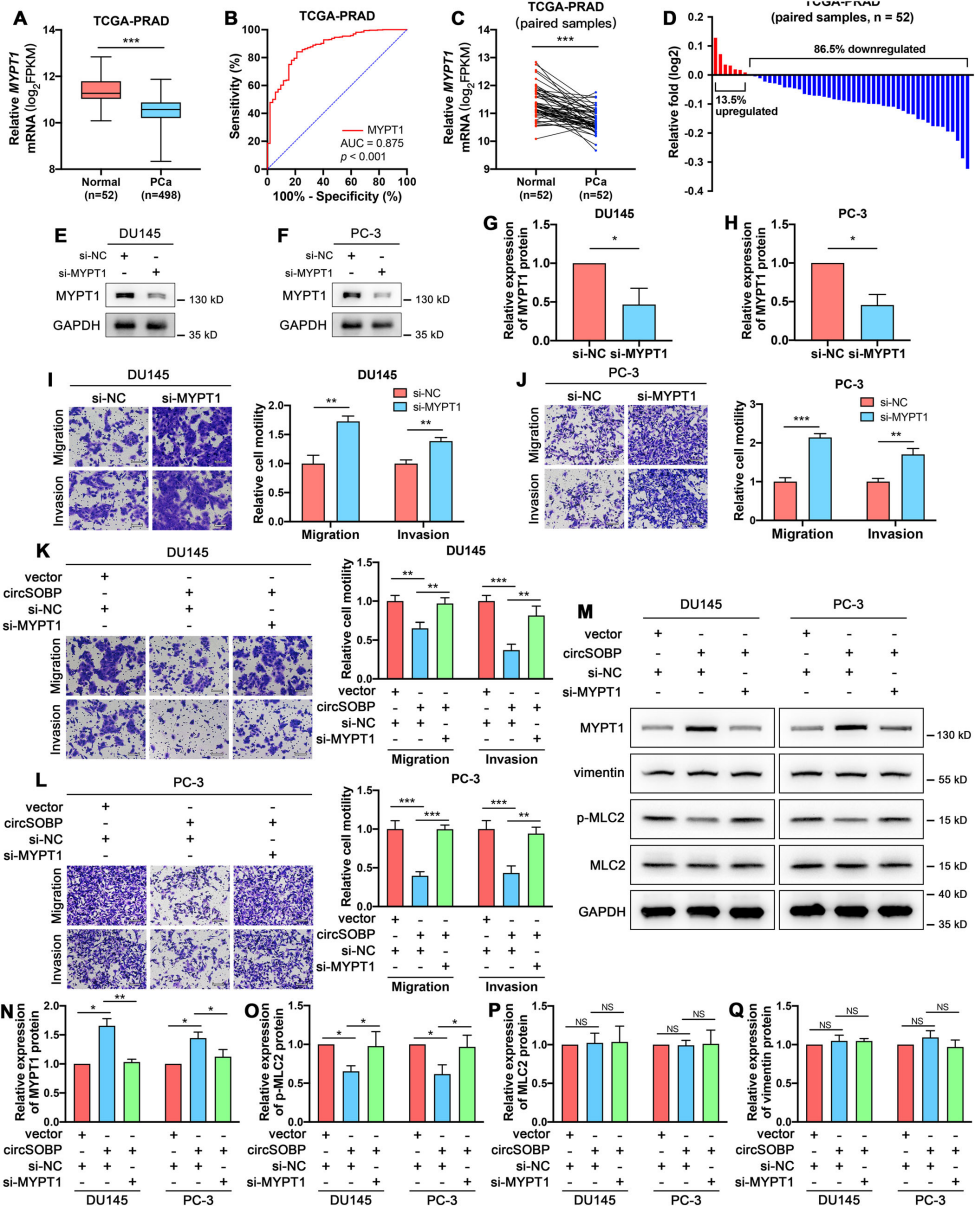
Depletion of MYPT1 reverses the inhibitory effect of circSOBP on PCa migration and invasion. (A) Expression of circSOBP in PCa and normal tissues from the TCGA database. Boxplot depicts median, 25th and 75th percentile, min‐max whiskers. ***P < .001, Mann–Whitney test. (B) The ROC curve in distinguishing PCa and ANP tissues by the expression of MYPT1. (C) Differential expression of circSOBP in 52 paired PCa and normal tissues from the TCGA database. ***P < .001, Wilcoxon matched‐pairs signed‐rank test. (D) The percentage of upregulated or downregulated expression of MYPT1 in 52 patients. (E) and (F) Efficacy of siRNA targeting MYPT1, analyzed using Western blot. (G) and (H) Densimetric analysis of the blots in (E) and (F), n = 3 independent experiments, GAPDH was used as a loading control. The data are presented as the mean ± SD. *P < .05, Student's *t*‐test, n = 3. (I) and (J) Effect of MYPT1 depletion on migration and invasion of DU145 and PC‐3 cell lines. The data are presented as the mean ± SD. **P < 0.01, ***P < .001, Student's *t*‐test, n = 3. (K) and (L) Effect of upregulated circSOBP accompanied by suppressing MYPT1 on migration and invasion of DU145 and PC‐3 cell lines. Scale bar, 100 μm. The data are presented as the mean ± SD. **P < .01, ***P < .001, one‐way ANOVA and Tukey's multiple comparisons test, n = 3. (M) Effect of upregulated circSOBP accompanied by suppressing MYPT1 on the expression of related proteins of DU145 and PC‐3 cells. (N)‐(Q) Densimetric analysis of the blots in (M), n = 3 independent experiments, GAPDH was used as the loading control. The data are presented as the mean ± SD. *P < .05, **P < .01, Student's *t*‐test. NC, normal control. NS, not significant

The data from the TCGA Research Network showed the low expression of MYPT1 in PCa (n = 52) compared with normal specimens (n = 498) (Figure 5A). The ROC curve indicated that the AUC of MYPT1 was 0.875 (cut‐off value = 11.020, sensitivity = 0.788, 1–specificity = 0.162) (Figure 5B), indicating that MYPT1 was a promising biomarker for distinguishing PCa tissues. MYPT1 was downregulated among the paired samples (n = 52) (Figure 5C). MYPT1 was downregulated in 45 (82.1%) PCa tissues compared with the paired normal specimens (Figure 5D).

MYPT1 was successfully depleted using siRNA (Figure 5E‐H). Migration and invasion abilities of DU145 (Figure 5I) and PC‐3 (Figure 5J) cells were enhanced after the depletion of MYPT1. The depletion of MYPT1 reversed the inhibitory effect of circSOBP on migration and invasion of both DU145 (Figure 5K) and PC‐3 (Figure 5L) cell lines. Furthermore, overexpression of circSOBP boosted MYPT1 expression, while inhibited p‐MLC2. The depletion of MYPT1 could reverse the regulatory effects of circSOBP on the expression of MYPT1 (Figure 5M,N) and p‐MLC2 (Figure 5M,O). The expression of total MLC2 (Figure 5M,P) and vimentin (Figure 5M,Q) was not affected by the change of circSOBP or MYPT1.

### 3.7 Flanking introns mediate the circularization of circSOBP

**FIGURE 6:**
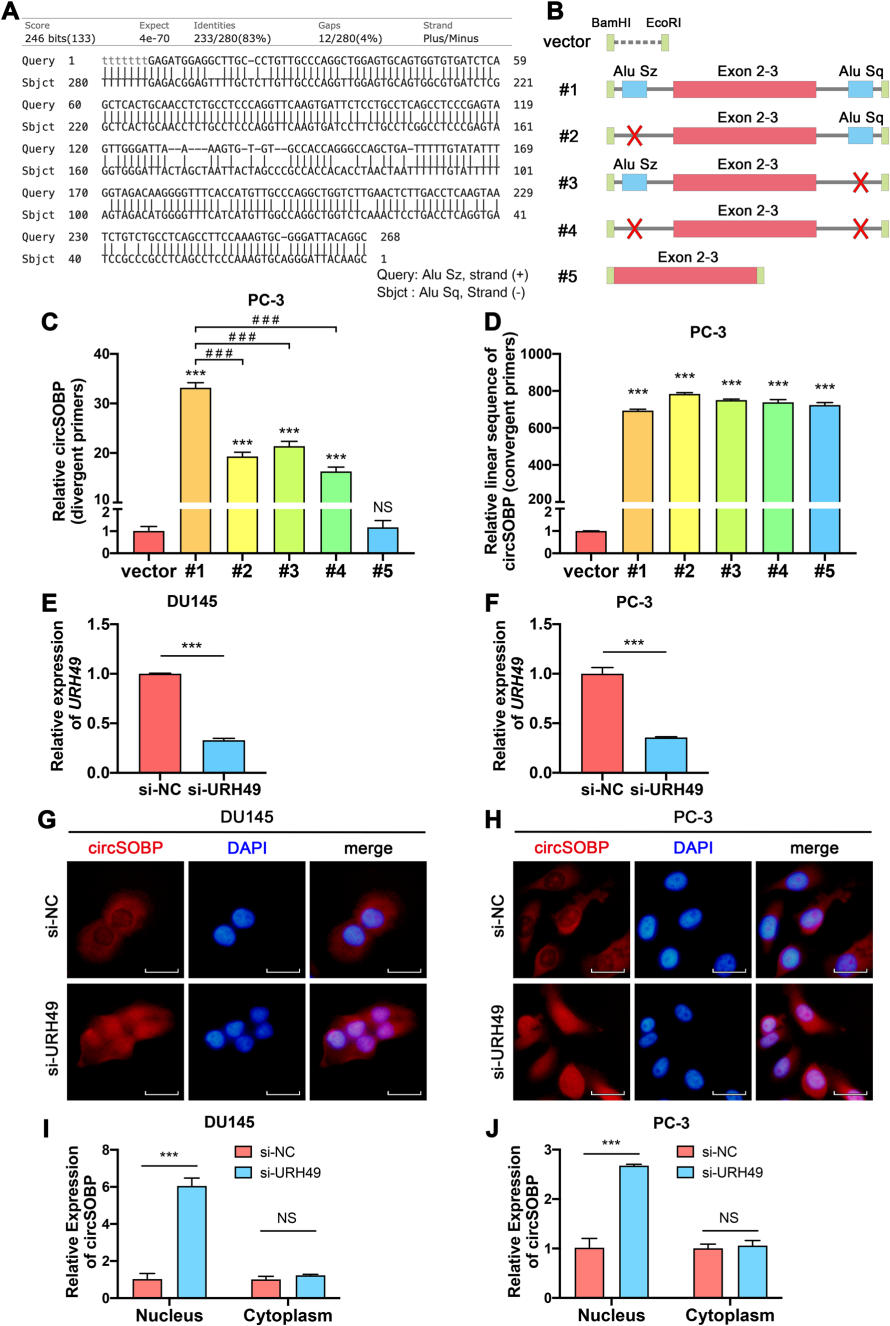
The circularization and nuclear export of circSOBP. (A) Alignment of Alu Sz and Alu Sq which were located on the flanking introns of circSOBP. (B) Schematic illustration of the plasmid constructions which were used to investigate the circularization of circSOBP. (C) Effect of the deletions of Alu sequences and flanking introns on the circularization of circSOBP analyzed using divergent primers. The data are presented as the mean ± SD. ***P < .001 comparing with vector, ###*P *< .001 comparing with #1, one‐way ANOVA and Dunnett's multiple comparisons test, n = 3. (D) Effect of the deletions of Alu sequences and flanking introns on the expression of the linear sequence of circSOBP analyzed using convergent primers. The data are presented as the mean ± SD. ***P < .001 comparing with vector, one‐way ANOVA, and Dunnett's multiple comparisons test, n = 3. (E) and (F) Efficacy of URH49 depletion using siRNA in DU145 and PC‐3 cell lines. The data are presented as the mean ± SD. *** *P *< .001, Student's *t*‐test, n = 3. (G) and (H) Effect of URH49 depletion on the subcellular distribution of circSOBP in DU145 and PC‐3 cells, analyzed using circRNA FISH assay. (I) and (J) Effect of URH49 depletion on the subcellular distribution of circSOBP in DU145 and PC‐3 cells, analyzed using qRT‐PCR. The data are presented as the mean ± SD. ***P < .001, Student's *t*‐test, n = 3. NC, normal control. NS, not significant

We found that there were two Alu elements, Alu Sz and Alu Sq, located in the flanking introns of circSOBP in the *SOBP* gene, which might boost the formation of circSOBP. By aligning the sequences of Alu Sz and Alu Sq using the Basic Local Alignment Search Tool (https://blast.ncbi.nlm.nih.gov/Blast.cgi), it was found that they were highly reverse complementary (Figure 6A). To verify whether the circularization of circSOBP was promoted by Alu Sz and Alu Sq, we constructed five sequences in pcDNA3.1 plasmid (Figure 6B): #1, Exon 2 & 3 with wild type of flanking introns containing the Alu elements; #2, Exon 2 & 3 and flanking introns without Alu Sz; #3, Exon 2 & 3 and flanking introns without Alu Sq; #4, Exon 2 & 3 and flanking introns without both Alu elements; #5, Exon 2 & 3 without flanking introns. After the transfection of the plasmids into PC‐3 cells, the qRT‐PCR assay was used to verify the circularization of circSOBP using divergent primers and convergent primers, respectively. Interestingly, we found that the flanking introns without the Alu elements (#2‐4) could also mediate the circularization of circSOBP (Figure 6C), while the #5 plasmid could only overexpress the linear sequence of circSOBP (Figure 6C,D). Nevertheless, #2‐4 plasmids overexpressed less circSOBP than #1 plasmid, probably due to the deletion of Alu elements (Figure 6C).

### 3.8 URH49 mediates the nuclear export of circSOBP

A previous study discovered that the depletion of URH49 caused short (<400 nucleotides) circRNAs to be enriched in the nucleus.^16^ Our findings indicated that circSOBP is a short circRNA (325 nucleotides) that mainly distributes in the cytoplasm. Therefore, we further explored whether URH49 could mediate the nuclear export of circSOBP. We successfully depleted the expression of URH49 in DU145 (Figure 6E) and PC‐3 (Figure 6F) cell lines using siRNA. CircRNA FISH assay (Figure 6G,H) and isolation of the nuclear and cytoplasmic RNA followed by qRT‐PCR (Figure 6I,J) suggested that URH49 depletion led to the accumulation of circSOBP in the nucleus.

## 4 DISCUSSION

The present study found a low expression of circSOBP in patients with PCa. CircRNAs are ubiquitously expressed in human tissues. More than 100 000 circRNAs have been identified, however, most of these molecules are understudied.^27^, ^39^ Previous research has reported a differential abundance of circRNAs in cancer tissues comparing with normal, a majority were downregulated in cancer.^39^ We have performed circRNA microarray using PCa tissues and ANP tissues from five patients to find differentially expressed circRNAs in PCa.^30^ After verification from PCa and ANP tissues from another 56 patients, we found circSOBP showed low expression in PCa. Besides, circSOBP expression was significantly correlated with the grade group of PCa patients, indicating that circSOBP might be related to the prognosis of patients with PCa. CircSOBP is derived from exon 2 and exon 3 of the *SOBP* gene, its function is unknown. We found that circSOBP governed PCa migration and invasion *in vitro* and metastasis *in vivo*. The depletion of circSOBP enhanced migration and invasion of PCa cells through regulating amoeboid migration. Amoeboid migration of cancer cells can lead to metastasis of cancer.

The invasion‐metastasis cascade^4^, ^40^, ^41^ envisions a succession of local invasion, intravasation, transit through the lymphatic and hematogenous systems, extravasation, formation of micrometastases, and colonization. Cancer cells metastasize through cell movement, mainly through mesenchymal migration and amoeboid migration. Cells undergoing mesenchymal migration express markers of EMT such as vimentin, E‐cadherin, N‐cadherin, etc.^42^ Cells that perform amoeboid migration require the interaction between p‐MLC2 and the F‐actin bundle to mediate the generation of high contractile forces, thereby, allowing cells to shuttle through the interstitial space.^43^, ^44^ Cells undergoing amoeboid migration could be distinguished by amoeboid pseudopods, cell roundness, and increased p‐MLC2.^7^, ^8^, ^9^ We discovered that the depletion of circSOBP led to an increase in p‐MLC2 expression, incidence of blebs, and stable blebs, and more roundness. Nevertheless, circSOBP did not shift the expression of vimentin. These results showed that circSOBP regulated amoeboid migration of PCa cells, but did not regulate the EMT‐mediated mesenchymal migration. This is the first study that discovers the relationship between circRNA and amoeboid migration of cancer.

We found that circSOBP inhibited migration and invasion by regulating amoeboid migration and altering the MYPT1/p‐MLC2 axis. CircSOBP could sponge miR‐141‐3p, thereby regulating MYPT1/p‐MLC2 expression. Previous studies have proved that individual circRNAs could act as a miRNA sponge.^32^, ^45^ For example, CDR1as, one of the most well‐characterized circRNAs, have conserved miRNA binding sites.^32^ CDR1as is resistant to miRNA‐mediated destabilization, but it sponges miR‐7 resulting in increased miR‐7 targets.^32^ We confirmed that circSOBP was located in the cytoplasm using a FISH assay, so it has the potential to function as a miRNA sponge. We found circSOBP could sponge miR‐141‐3p, thereby upregulate MYPT1 expression, which is targeted by miR‐141‐3p. MYPT1 is a subunit of myosin phosphatase which mediates the dephosphorylation of p‐MLC2,^37^, ^38^ while p‐MLC2 interacts with F‐actin and produces the contractile force for cell movement.^43^, ^44^

Complementary sequences in the genomic flanking introns could facilitate the circularization of circRNAs.^11^, ^12^, ^13^ In human cells, most flanking intronic complementary sequences are Alu elements.^11^, ^12^ Previous studies found that the deletion of Alu elements significantly reduces the circularization of circRNAs.^12^, ^46^ We found that there were two reverse complementary Alu elements in the flanking introns of circSOBP. However, flanking introns with the deletion of Alu elements could also mediate the circularization of circSOBP, although the production of circSOBP was reduced by comparing with the wild‐type introns. This finding indicates that complementary Alu elements are not necessary for the formation of circSOBP. A possible explanation for this might be that RNA binding protein could bind to the flanking introns and boost the formation of circRNAs.^47^, ^48^, ^49^ The knockout of circRNAs using CRISPR‐Cas9‐mediated deletion of Alu elements^50^ might be unreliable for certain circRNAs.

## 5 CONCLUSIONS

**FIGURE 7:**
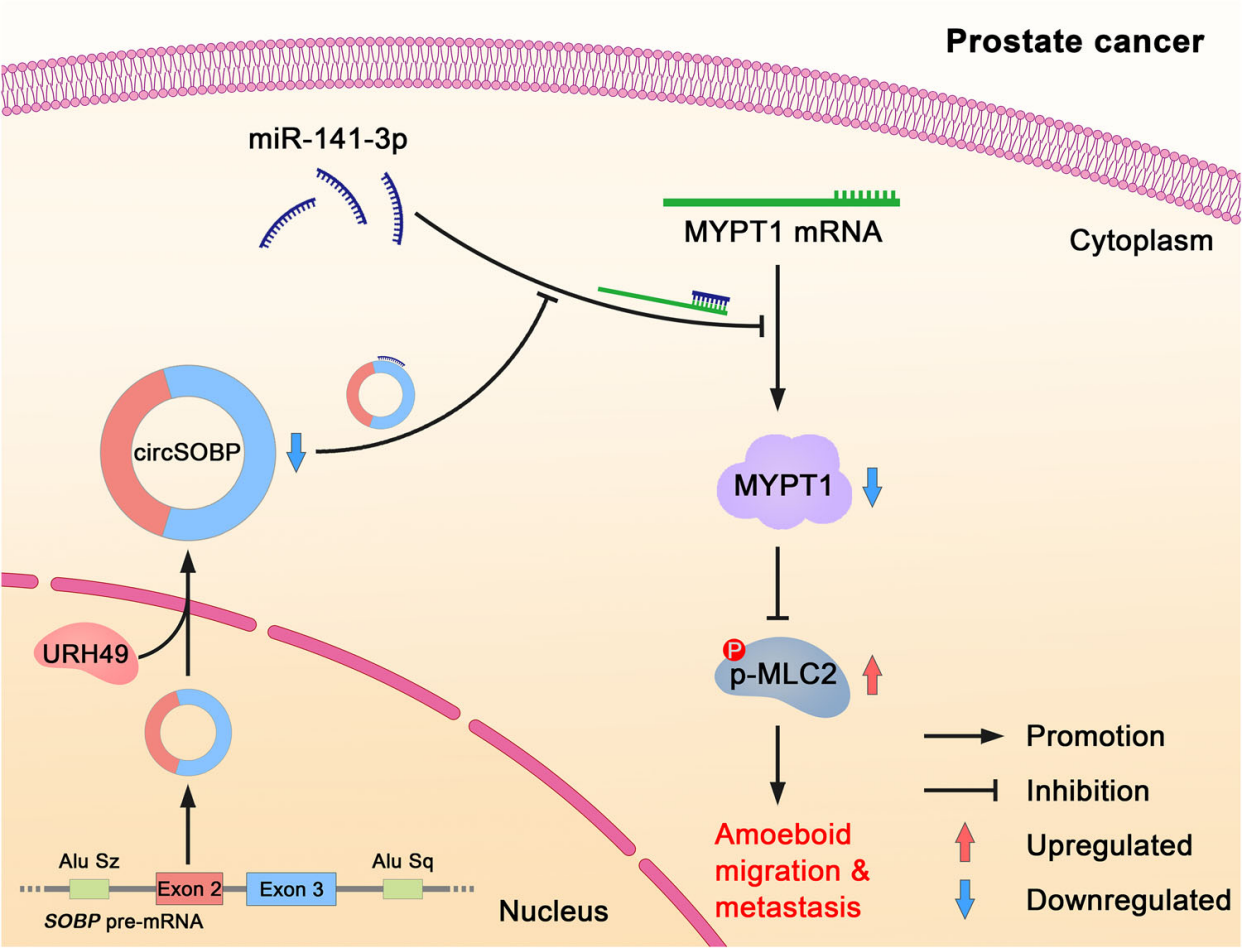
Schematic illustration of the mechanism of circSOBP regulating amoeboid migration of PCa cells. CircSOBP functioned as a sponge for miR‐141‐3p, and in turn upregulated the expression of MYPT1, which dephosphorylate p‐MLC2. This pathway governed the amoeboid migration of PCa cells

The present study identified a novel circRNA circSOBP that was downregulated in PCa. Overexpression of circSOBP suppressed PCa cells’ migration and invasion *in vitro* and metastasis *in vivo*. CircSOBP depletion increased migration and invasion and induced amoeboid migration of PCa cells. The inhibitory effect of circSOBP on PCa cell migration and invasion was at least in part due to the regulation of the miR‐141‐3p/MYPT1/p‐MLC2 axis. Complementary intronic Alu elements could promote but would not be necessary for the formation of circSOBP. The nuclear export of circSOBP was mediated by URH49 (Figure 7). Our study discovered the role of circSOBP and the mediation of amoeboid migration in cancer cells for the first time, which may provide a promising target for the therapeutic application in patients with PCa.

## ETHICS APPROVAL AND CONSENT TO PARTICIPATE

The present study was approved by the Ethics Committee of Jinshan Hospital, Fudan University. Written consent was obtained from each participant.

## CONFLICT OF INTEREST

The authors declare that they have no conflict of interest.

## Supporting information

Supporting information

Supporting information

Supporting information

Supporting information

Supporting information

Supporting information

Supporting information

Supporting information

## Data Availability

The datasets used and/or analyzed during the current study are available from the corresponding author on reasonable request.
