# Supplementary material for: Novel circular RNA circSOBP governs amoeboid migration through the regulation of the miR‐141‐3p/MYPT1/p‐MLC2 axis in prostate cancer
Source: Clin Transl Med. 2021 Mar 26;11(3):e360. doi: 10.1002/ctm2.360 (PMC8002909; doi:10.1002/ctm2.360)
Supplement: Supplementary file 1 — Supporting information [file CTM2-11-e360-s008.docx]

**
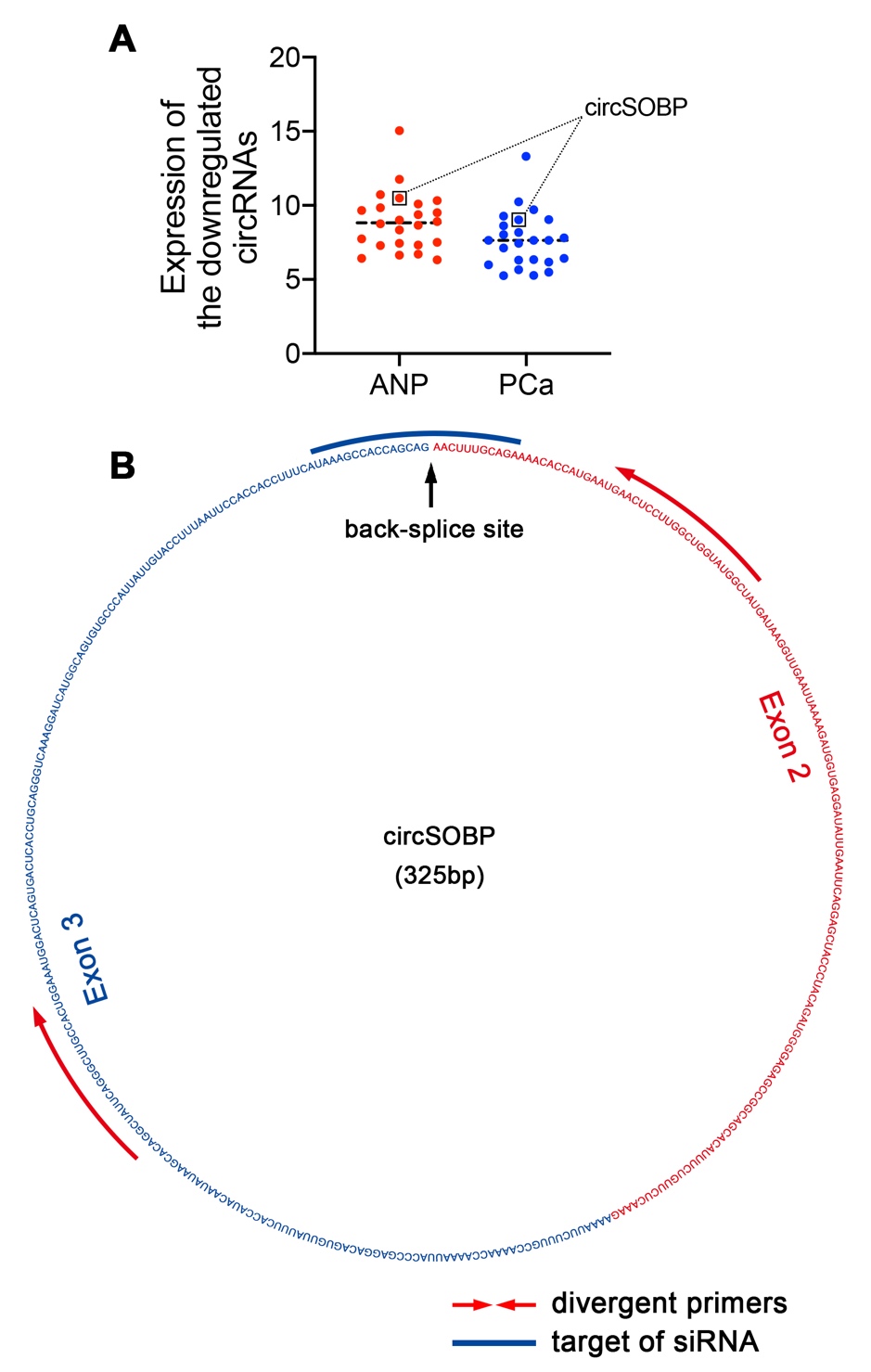
**

**Additional Figure S1** The abundance and sequence of circSOBP. (A) Scatter plot of the significantly downregulated circRNAs in the microarray. Each dot indicates 1 circRNA. The black dotted line indicates the median. (B) The sequence of circSOBP. The black arrow indicates the back-splice junction of circSOBP. The red arrows indicate the divergent primers for PCR. The blue line indicates the target of siRNA.
