# Supplementary material for: Novel circular RNA circSOBP governs amoeboid migration through the regulation of the miR‐141‐3p/MYPT1/p‐MLC2 axis in prostate cancer
Source: Clin Transl Med. 2021 Mar 26;11(3):e360. doi: 10.1002/ctm2.360 (PMC8002909; doi:10.1002/ctm2.360)
Supplement: Supplementary file 2 — Supporting information [file CTM2-11-e360-s001.docx]

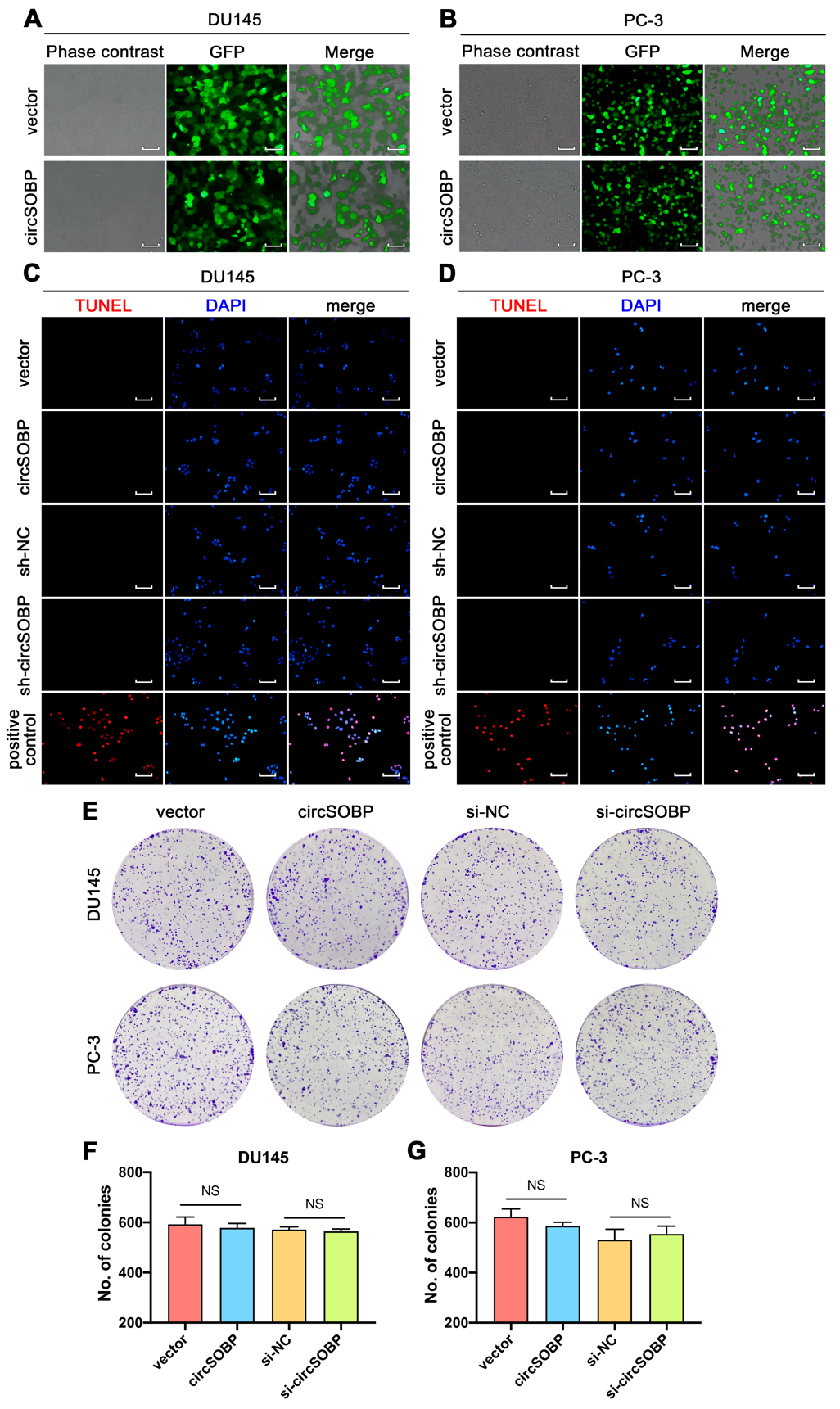


**Additional Figure S2** Effects of forced circSOBP expression on apoptosis and colony formation of PCa cells. (A)-(B) Lentivirus infected DU145 and PC-3 cells expressed green fluorescence protein (GFP). Scale bar, 100μm. (C)-(D) Effects of overexpressing or depleting circSOBP on apoptosis of DU145 and PC-3 cells, analyzed using TUNEL staining. Scale bar, 100μm. (E) Effects of overexpressing or depleting circSOBP on colony formation of DU145 and PC-3 cells, representative images. (F)-(G) Quantitative analyses of the colonies in (E). The data are presented as the mean ± SD. Student’s *t* test, n=3. NS, not significant.
