# Supplementary material for: Novel circular RNA circSOBP governs amoeboid migration through the regulation of the miR‐141‐3p/MYPT1/p‐MLC2 axis in prostate cancer
Source: Clin Transl Med. 2021 Mar 26;11(3):e360. doi: 10.1002/ctm2.360 (PMC8002909; doi:10.1002/ctm2.360)
Supplement: Supplementary file 3 — Supporting information [file CTM2-11-e360-s004.docx]

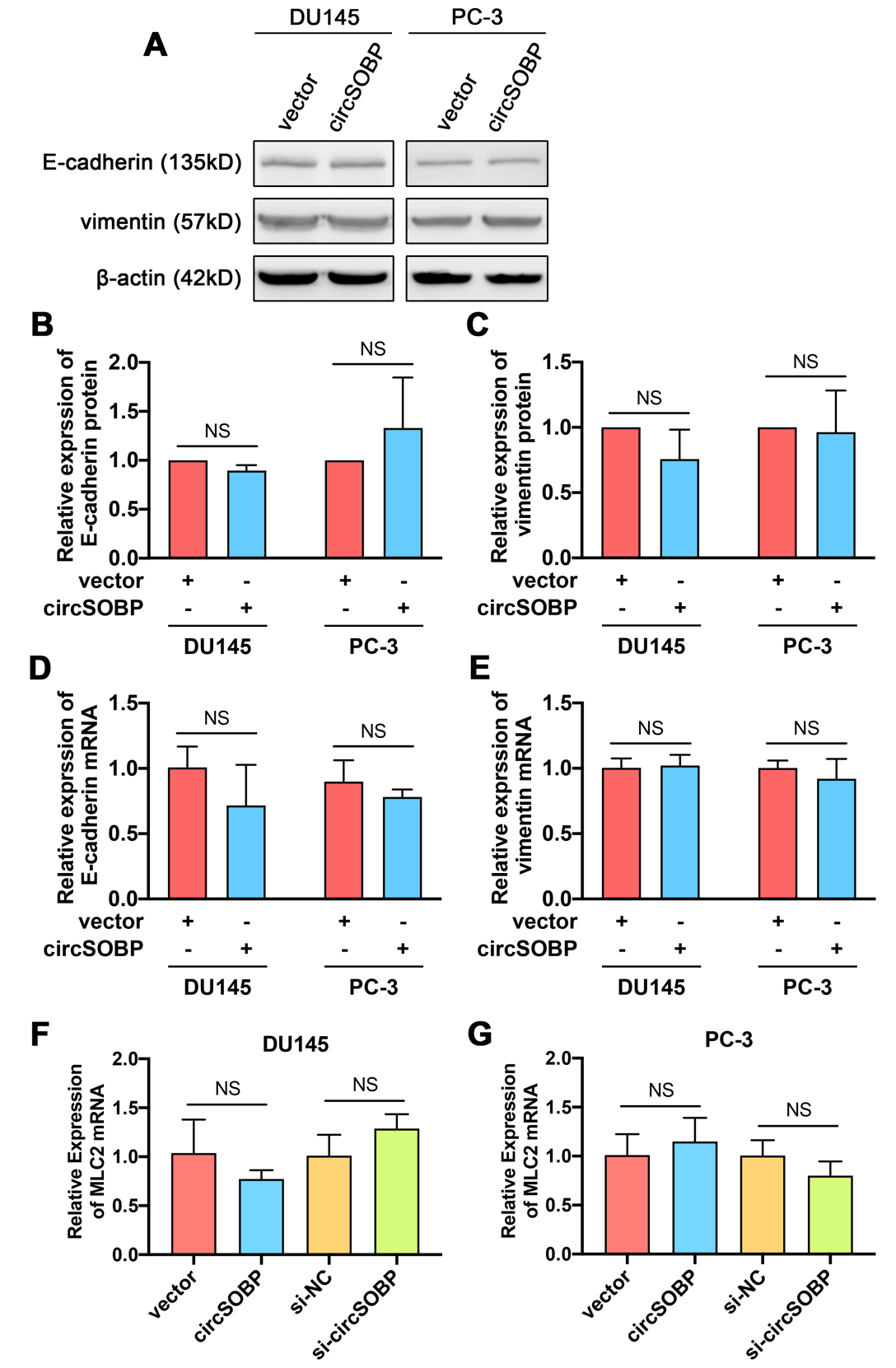


**Additional Figure S3** Effects of overexpressing circSOBP on the expression of E-cadherin, vimentin and MLC2. (A) Effect of overexpressing circSOBP on the expression of E-cadherin and vimentin in DU145 and PC-3 cells, analyzed using Western blot. (B)-(C) Densimetric analysis of the blots in (A), β-actin was used as a loading control. The data are presented as the mean ± SD. Student’s *t* test, n=3. (D)-(E) Effect of overexpressing circSOBP on the expression of E-cadherin (*CAD1*) and vimentin (*VIM*) mRNA in DU145 and PC-3 cells. The data are presented as the mean ± SD. Student’s *t* test, n=3. (F)-(G) Effect of circSOBP overexpression and depletion on the expression of MLC2 mRNA in DU145 and PC-3 cells. The data are presented as the mean ± SD. Student’s *t* test, n=3. NS, not significant.
