# Supplementary material for: Novel circular RNA circSOBP governs amoeboid migration through the regulation of the miR‐141‐3p/MYPT1/p‐MLC2 axis in prostate cancer
Source: Clin Transl Med. 2021 Mar 26;11(3):e360. doi: 10.1002/ctm2.360 (PMC8002909; doi:10.1002/ctm2.360)
Supplement: Supplementary file 4 — Supporting information [file CTM2-11-e360-s007.docx]

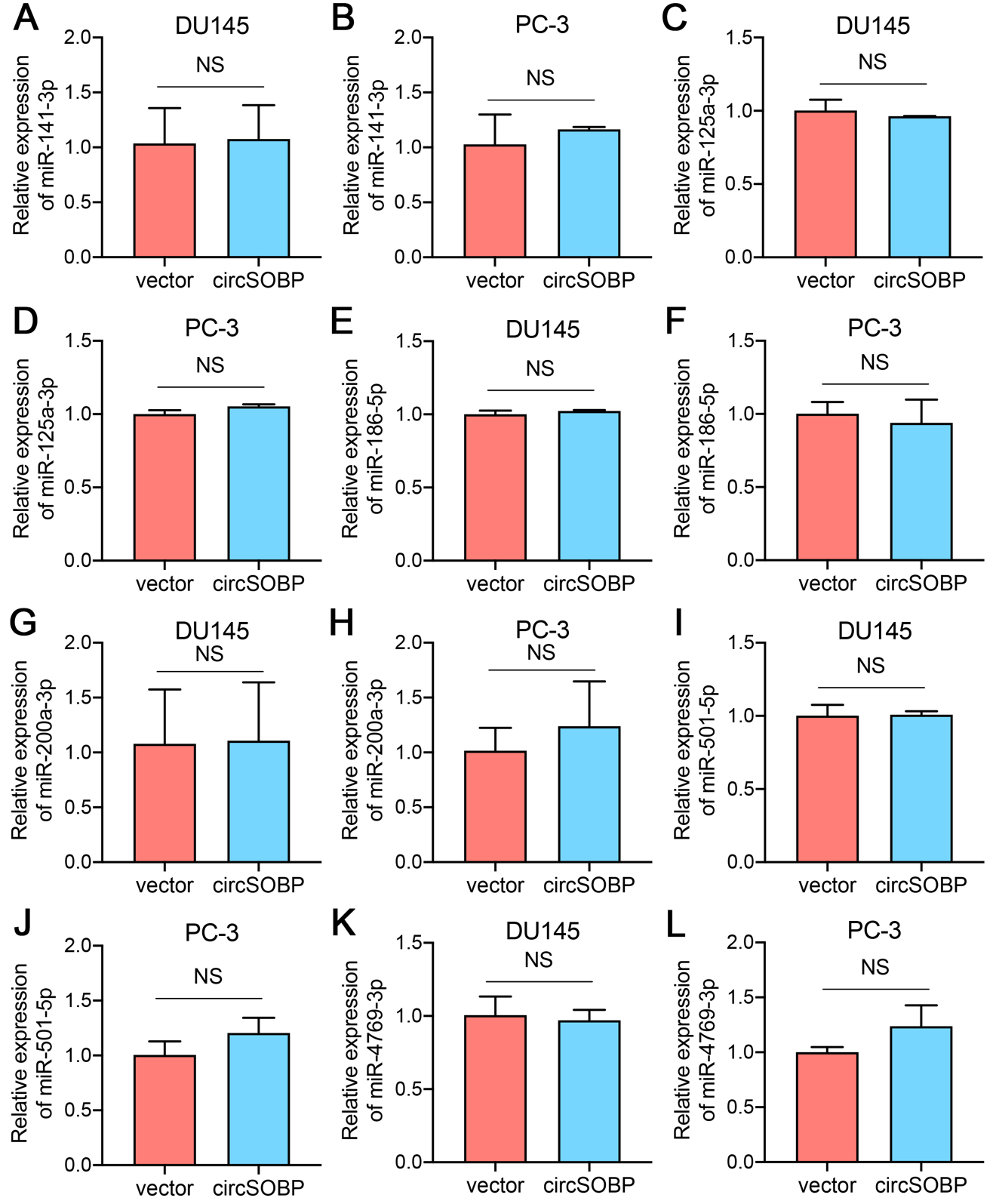


**Additional Figure S4** Effect of overexpressing circSOBP on the expression of predicted miRNAs. (A-B) Effect of overexpressing circSOBP on miR-141-3p expression of DU145 and PC-3 cells. (C-D) Effect of overexpressing circSOBP on miR-125a-3p expression of DU145 and PC-3 cells. (E-F) Effect of overexpressing circSOBP on miR-186-5p expression of DU145 and PC-3 cells. (G-H) Effect of overexpressing circSOBP on miR-200a-3p expression of DU145 and PC-3 cells. The data are presented as the mean ± SD. Student’s *t* test, n=3. (I-J) Effect of overexpressing circSOBP on miR-501-5p expression of DU145 and PC-3 cells. (K-L) Effect of overexpressing circSOBP on miR-4769-3p expression of DU145 and PC-3 cells. The data are presented as the mean ± SD. Student’s *t* test, n=3. NS, not significant.
