# Supplementary material for: Novel circular RNA circSOBP governs amoeboid migration through the regulation of the miR‐141‐3p/MYPT1/p‐MLC2 axis in prostate cancer
Source: Clin Transl Med. 2021 Mar 26;11(3):e360. doi: 10.1002/ctm2.360 (PMC8002909; doi:10.1002/ctm2.360)
Supplement: Supplementary file 5 — Supporting information [file CTM2-11-e360-s006.docx]

**Supplementary Table S1. The sequences of siRNAs.**

| **Gene/circRNA** | **Gene ID/circBase ID** | **Sense (5’-3’)** | **Antisense (5’-3’)** |
| --- | --- | --- | --- |
| circSOBP | hsa_circ_0001633 | AGCCACCAGCAGAACUUUGCATT | UGCAAAGUUCUGCUGGUGGCUTT |
| MYPT1 | 4659 | GCAGCUCGAAAGGAAGAAGAATT | UUCUUCUUCCUUUCGAGCUGCTT |
| URH49 | 10212 | AAAGGCCUAGCCAUCACUUUU | AAAAGUGAUGGCUAGGCCUUU |

Gene IDs are from <https://www.ncbi.nlm.nih.gov/gene>; circBase IDs are from <http://www.circbase.org>.
