# Supplementary material for: Novel circular RNA circSOBP governs amoeboid migration through the regulation of the miR‐141‐3p/MYPT1/p‐MLC2 axis in prostate cancer
Source: Clin Transl Med. 2021 Mar 26;11(3):e360. doi: 10.1002/ctm2.360 (PMC8002909; doi:10.1002/ctm2.360)
Supplement: Supplementary file 6 — Supporting information [file CTM2-11-e360-s003.docx]

**Supplementary Table S2. The sequences of primers for qRT-PCR.**

| **Gene/circRNA** | **Gene ID/circBase ID** | **Forward primer**  **(5’-3’)** | **Reverse primer**  **(5’-3’)** | **Product size (nt)** |
| --- | --- | --- | --- | --- |
| circSOBP  divergent | hsa_circ_0001633 | GGCTATTCAGGGCTTGCCAC | GCCATACCAGCCAAGGAGTTC | 166 |
| circSOBP convergent | hsa_circ_0001633 | TGCCAAAACCAAAATTACCC | ACTGCCATGATCCTTTGACC | 125 |
| SOBP | 55084 | AGACCTCCCGAAAATAAACGGA | CATCTGTAGGGTAGCTCCTGAAT | 178 |
| MYPT1 | 4659 | AGTTAATCGGCAAGGGGTTGA | ATGACCACTATTTAGCCACTGC | 94 |
| URH49 | 10212 | GCCCCAGGCTCCTCAAGAGA | CTCATGCTGGACCTCAGAAG | 166 |
| E-cadherin (CDH1) | 999 | CGAGAGCTACACGTTCACGG | GGGTGTCGAGGGAAAAATAGG | 119 |
| vimentin (VIM) | 7431 | AGTCCACTGAGTACCGGAGAC | CATTTCACGCATCTGGCGTTC | 98 |
| MLC2 | 4633 | TTGGGCGAGTGAACGTGAAAA | CCGAACGTAATCAGCCTTCAG | 194 |
| GAPDH | 2597 | ACAACTTTGGTATCGTGGAAGG | GCCATCACGCCACAGTTTC | 101 |

Gene IDs are from <https://www.ncbi.nlm.nih.gov/gene>; circBase IDs are from <http://www.circbase.org>; nt, nucleotides.
