# Supplementary material for: Novel circular RNA circSOBP governs amoeboid migration through the regulation of the miR‐141‐3p/MYPT1/p‐MLC2 axis in prostate cancer
Source: Clin Transl Med. 2021 Mar 26;11(3):e360. doi: 10.1002/ctm2.360 (PMC8002909; doi:10.1002/ctm2.360)
Supplement: Supplementary file 7 — Supporting information [file CTM2-11-e360-s005.docx]

**Supplementary Table S3. The antibodies used in the present study.**

| **Antibody** | **Dilution** | **Company** | **Cat No.** |
| --- | --- | --- | --- |
| E-cadherin rabbit mAb | 1:2000 (WB) | Cell Signaling Technology | 3195 |
| Vimentin rabbit mAb | 1:2000 (WB) | Cell Signaling Technology | 5741 |
| MYPT1 rabbit mAb | 1:2000 (WB) | Cell Signaling Technology | 8574 |
| MLC2 rabbit mAb | 1:2000 (WB) | Cell Signaling Technology | 8505 |
| p-MLC2 rabbit antibody | 1:2000 (WB)  1:50 (IF) | Cell Signaling Technology | 3671 |
| GAPDH HRP-conjugated mouse mAb | 1:2000 (WB) | Proteintech | HRP-60004 |
| β-actin monoclonal antibody | 1:5000 (WB) | Proteintech | 66009-1-Ig |
| anti-rabbit IgG HRP linked antibody | 1:5000 (WB) | Cell Signaling Technology | 7074 |
| anti-mouse IgG HRP linked antibody | 1:5000 (WB) | Cell Signaling Technology | 7076 |
| Alexa Fluor 555-labeled donkey  anti-rabbit IgG | 1:1000 (IF) | Beyotime | P0179 |

WB, western blotting; IF, Immunofluorescence; HRP, horseradish peroxidase.
