# Supplementary material for: Novel circular RNA circSOBP governs amoeboid migration through the regulation of the miR‐141‐3p/MYPT1/p‐MLC2 axis in prostate cancer
Source: Clin Transl Med. 2021 Mar 26;11(3):e360. doi: 10.1002/ctm2.360 (PMC8002909; doi:10.1002/ctm2.360)
Supplement: Supplementary file 8 — Supporting information [file CTM2-11-e360-s002.docx]

**Supplementary Table S4. Sequences of the FISH probes.**

| **Target** | **Label** | **Sequence (5’-3’)** |
| --- | --- | --- |
| circSOBP-1 | 5’ Cy3 | TGTTTTCTGCAAAGTTCTGCTGGTG |
| circSOBP-2 | 5’ Cy3 | GCAAAGTTCTGCTGGTGGCTTTATG |
| circSOBP-3 | 5’ Cy3 | GTTCTGCTGGTGGCTTTATGAAAGG |
